# Dangerousness assessment in psychiatric inpatients suffering from psychotic disorders

**DOI:** 10.1192/j.eurpsy.2021.1012

**Published:** 2021-08-13

**Authors:** S. Khouadja, R. Melki, C. Ben Taleb, L. Zarrouk

**Affiliations:** 1 Psychiatry, University Hospital Of Mahdia., Mahdia, Tunisia; 2 Psychiatry, University Hospital Of Mahdia, Mahdia, Tunisia

**Keywords:** psychiatry, dangerousness, schizophrénia, Psychostic disorders

## Abstract

**Introduction:**

Dangerousness is a state in which a person is likely to commit violent acts.

**Objectives:**

Describe the socio-demographic and clinical characteristics of psychiatric inpatients hospitalized in the locked unit and suffering from schizophrenia or other psychotic disorders and to assess their dangerousness.

**Methods:**

This is a cross-sectional study carried out in the locked unit of psychiatric department of the University Hospital of Mahdia during one year. We have collected data of patients diagnosed with schizophrenia or other psychotic disorders according to DSM 5. Psychometric assessment was done using the BPRS, the PANSS, the VRAG and the HCR-20 scales.

**Results:**

We have included 173 patients. The average age was 36 years with a sex ratio of 9. The majority of our patients were unmarried and of a low economic level. Alcohol and cannabis consumption was found in 7.6% and in 5.7% of cases respectively. A history of incarceration was found in 79% of cases. Homicide was the infraction the most committed in 8% of cases. 71.2% of patients had an anterior hospitalization in the locked unit. Aggressiveness and instability were the main indication for hospitalization. The diagnosis was schizophrenia in 84% of cases. Patients were treated with classic antipsychotic drugs in 55.8% of cases. Non-adherence to treatment was reported in 33% of cases. The average score of psychometric scales were BPRS = 21.4; VRAG = - 4.87 and HCR-20 = 17± 0.87.

**Conclusions:**

Our study showed comparable assessments for dangerousness with the literature. Evaluating dangerousness should represent the first step of the therapeutic process.

